# Integration of single-cell RNA sequencing and bulk RNA sequencing to reveal an immunogenic cell death-related 5-gene panel as a prognostic model for osteosarcoma

**DOI:** 10.3389/fimmu.2022.994034

**Published:** 2022-09-26

**Authors:** Jiaqi Yang, Jian Zhang, Song Na, Zhizhou Wang, Hanshuo Li, Yuxin Su, Li Ji, Xin Tang, Jun Yang, Lu Xu

**Affiliations:** ^1^ Department of Dermatology, Second Affiliated Hospital of Dalian Medical University, Dalian, China; ^2^ Department of Orthopedics, First Affiliated Hospital of Dalian Medical University, Dalian, China; ^3^ Emergency Intensive Care Unit, Affiliated Zhongshan Hospital of Dalian University, Dalian, China; ^4^ Department of General Surgery, First Affiliated Hospital of Dalian Medical University, Dalian, China; ^5^ Cardiovascular Research Institute of Northern Theater Command General Hospital, Shenyang, China; ^6^ Department of Gastroenterology, DongZhiMen Hospital, Beijing University of Chinese Medicine, Beijing, China; ^7^ Institute of Cancer Stem Cell, Dalian Medical University, Dalian, China

**Keywords:** osteosarcoma, immunogenic cell death, prognostic panel, molecular subtype, single cell RNA sequencing, bulk RNA sequencing

## Abstract

**Background:**

Despite the comparatively low prevalence of osteosarcoma (OS) compared to other cancer types, metastatic OS has a poor overall survival rate of fewer than 30%. Accumulating data has shown the crucial functions of immunogenic cell death (ICD) in various cancers; nevertheless, the relationship between ICD and OS was not previously well understood. This research aims to determine the function of ICD in OS and construct an ICD-based prognostic panel.

**Methods:**

Single cell RNA sequencing data from GSE162454 dataset distinguished malignant cells from normal cells in OS. The discrepancy in ICD scores and corresponding gene expression was intensively explored between malignant cells and normal cells. Using the RNA sequencing data of the TARGET-OS, GSE16091, GSE21257, and GSE39058 datasets, the molecular subtype of OS was determined by clustering seventeen ICD-related genes obtained from the literature. Differentially expressed genes (DEGs) between different molecular subtypes were identified to develop a novel ICD-associated prognostic panel.

**Results:**

The malignant cells had a remarkable decrease in the ICD scores and corresponding gene expression compared with normal cells. A total of 212 OS patients were successfully stratified into two subtypes: C1 and C2. C1-like OS patients were characterized by better prognostic outcomes, overexpression of ICD genes, activation of the ICD pathway, high inflitration abundance of immunocytes, and low expression levels of immune checkpoint genes (ICGs); however, the reverse is true in C2-like OS patients. Utilizing the limma programme in R, the DEGs between two subtypes were determined, and a 5-gene risk panel consisting of BAMBI, TMCC2, NOX4, DKK1, and CBS was developed through LASSO-Cox regression analysis. The internal- and external-verification cohorts were employed to verify the efficacy and precision of the risk panel. The AUC values of ROC curves indicated excellent prognostic prediction values of our risk panel.

**Conclusions:**

Overall, ICD represented a protective factor against OS, and our 5-gene risk panel serving as a biomarker could effectively evaluate the prognostic risk in patients with OS.

## Introduction

Osteosarcoma (OS) is a primary malignant bone tumor that affects mostly children and teenagers (1, 2). OS is known as malignant tumor, which often occurs in the metaphysis of long bones, including the arms, legs, knees, and shoulders, and is distinguished by a poor prognosis and a high incidence of impairment (3, 4). Multimodal treatment has improved these patients’ 5-year survival rates to about 70%, particularly when neoadjuvant chemotherapy is used in conjunction with extensive surgical resection (5). Nevertheless, a significant proportion of individuals present with metastases when initial diagnosis or following intense therapy (6, 7). More than half of these individuals will die within five years (6, 7). It is thus imperative that indicators of osteosarcoma’s biological heterogeneity be identified in order to enhance prognosis.

Cell death has been defined and interpreted from morphological, biochemical, and functional viewpoints by the Nomenclature Committee on Cell Death throughout the last decade (8). Immunogenic cell death (ICD) was initially hypothesized in the context of anticancer treatment, and was based on animal studies that revealed that tumor-specific immune responses might decide the success of anticancer medicines (9). The ICD is aimed to stimulate the immune system in immunocompetent hosts. When ICD occurs, a slew of damage-associated molecular patterns (DAMPs) are exposed and released, giving dying cancer cells a powerful adjuvanticity boost by attracting and activating antigen-presenting cells (10–12). Diverse innate immune receptors are implicated in DAMPs-mediated ICD, and their collaboration with DAMPs is required for ICD and anti-tumor immune response (13). However, the therapeutic potential and mechanism of harnessing ICD in OS have not yet been thoroughly studied. Therefore, the in-depth understanding of the correlation between ICD-related genes and overall survival of OS maybe invent a novel method for the therapy and prognosis evaluation in patients with OS.

In this research, a molecular classifier of OS was successfully established depending on the expression profiles of ICD-related genes in the TARGET-OS, GSE16091, GSE21257, and GSE39058 datasets. The relationship between molecular clusterss, prognosis, immunocyte inflitration, and ICD activity was explored. A robust 5-gene risk panel that contained BAMBI, TMCC2, NOX4, DKK1, and CBS was developed using differentially expressed genes (DEGs) between the OS subtypes, which could serve as a biomarker to effectively evaluate the prognostic risk in patients with OS.

## Methods

### Data collection and processing

Single-cell RNA sequencing (scRNA-seq) data GSE162454 was downloaded from GEO platform, which detected a total of 6 osteosarcoma tissues based on the 10X Genomics (14). RNA-sequencing (RNA-seq) data and corresponding follow-up information of TARGET-OS samples were obtained from the TARGET database (https://ocg.cancer.gov/programs/target) (15, 16). The expression profiles and clinical information in the GSE16091, GSE21257, and GSE39058 datasets were acquired from the GEO database (https://www.ncbi.nlm.nih.gov/geo/) (17–19). In total, there were 17 ICD-related genes collected from the literatures (20, 21).

The detailed processing of scRNA-seq data was illustrated below: 1) The ‘Seurat’ package was used to preprocess the scRNA-seq data (22); the PercentageFeatureSet function was used to determine the proportion of mitochondrial genes; and correlation analysis was utilized to investigate the relationship between sequencing depth and mitochondrial gene sequences and/or total intracellular sequences. 2) Set each gene to be expressed in at least 5 cells. 3) The expression of genes in each cell is more than 300 and less than 5000, the content of mitochondria is less than 10%, and the UMI of each cell is at least greater than 1000 were preserved. 3) The scRNA-seq data were normalized by the LogNormalize method after data filtering.

The detailed processing of RNA-seq data of TARGET-OS cohort was illustrated below: 1) The samples that lacked of corresponding follow-up information were eliminated; 2) The Gene Symbol format was obtained by converting the ENSEMBL gene IDs; 3) The median value was computed using multiple Gene Symbol expressions.

The detailed processing of microarray data of GEO-OS cohort was illustrated below: 1) The samples that lacked of corresponding follow-up information were eliminated; 2) The Gene Symbol format was obtained by converting the probe IDs; 3) Probes were removed because of their correspondences to multiple genes; 4) The average value was regarded as the gene expression while multiple probes were corresponded to one gene.

The intersecting genes were acquired *via* taking the intersection between the mRNA expression profiles from TARGET and GEO datasets. Using the “ComBat” function from the “sva” package in R, the expression data of intersecting genes were transformed into log2(x + 1) format and batch normalised (23, 24). The mRNA expression profiles of these intersecting genes were curated with corresponding follow-up data in TARGET and GEO datasets, respectively. After preprocessing, we enrolled 84 OS samples from TARGET-OS, 34 OS samples from GSE16091 dataset, 53 OS samples from GSE21257 dataset, and 41 OS samples from GSE39058 dataset.

### Potential regulatory pathways between tumor cells and normal cells

The tSNE dimensionality reduction of 28968 cells were performed using “RunTSNE” functions in R. Subsequently, the “CellCycleScoring” function was employed to calculate S and G2M scores based on S phase and G2M phase gene expression, and predicts classification of each cell in either S, G2M, or the G1 phase. Meanwhile, the “copykat” package in R was applied to predict copy number variation of each cell, and in turn, infer diploid (normal cells) and aneuploid (tumor cells) (25).

Following these, we downloaded and curated 50 typical hallmark pathways and ICD pathway from the Molecular Signatures Database (MsigDB, http://www.gsea-msigdb.org/gsea/index.jsp) (26, 27). Through ssGSEA analysis of tumor cells and normal cells in each sample, we obtained the enrichment score of each pathway. A heatmap was utilized to show the discrepancies of pathway enrichment scores and ICD-related gene expressions between diploid (normal cells) and aneuploid (tumor cells).

### Consistency clustering algorithm, gene set variation analysis and gene set enrichment analysis

From 212 OS samples, the expression patterns and clinical data of 17 ICD-related genes were derived. Next, these ICD-related genes were clustered using ConsensusClusterPlus (parameters: reps = 50, pItem = 0.8, pFeature = 1, clusterAlg = “km”, distance=“euclidean”) (28–30). The km and euclidean distances were used as a clustering algorithm and distance measure, respectively. The “GSVA” package in R was applied to compute the ICD scores of each patient with OS, which could serve as the indicator of ICD activites (31, 32). The “wilcox.test” fucntion in R was then employed to compare the discrepancy in the ICD scores between different clusters. In addition to ICD scores, the enrichment scores of 50 typical HALLMARKER-signaling pathways were also computed by “GSVA” package; meanwhile, similar method was applied to compare the potential discrepancy of signaling pathways between different clusters.

### Cluster-based analysis of tumor immune microenvironment

The “estimate” package in R was employed to compute the ImmuneScore, StromalScore, EstimateScore, and tumor purity of each OS sample, and the “ggpubr” package in R was used to visualize this result (33). In addition, the TIMER2.0 database (http://timer.cistrome.org/) provides a complete immunological signature of tumor infiltrating cells in a variety of tumor samples from the TCGA database on the basis of the algorithms of TIMER, CIBERSORT, EPIC, and MCPCOUNTER (34). The ‘pheatmap’ package in R was used to illustrate the infiltration of distinct immune cells in each OS sample. Subsequently, the ‘limma’ package in R was used to determine statistically significant changes in immune cell infiltration between C1 and C2 subgroups; cells were then isolated and stored based on these p values (p < 0.05).

The activation of immune checkpoint genes (ICGs) that suppress antitumor immune responses is crucial for the immunosurveillance evading and malignant progression of tumor cells. It has been generally accepted that ICGs played an irreplaceable role in regulating the functions of immunocytes and disease progression. Thus, we further explored the discrepancy of the ICGs expression levels between C1 and C2 populations.

### Identification of differentially expressed genes

The “limma” programme was utilized to determine the DEGs between C1 and C2 subtypes, and the filtering thresholds were as follows: 1) FDR < 0.05; 2) fold-change (FC) > 1.5 or FC <2/3. Then, the identified DEGs were investigated by performing Kyoto Encyclopedia of Genes and Genomes (KEGG) pathway analysis and Gene Ontology (GO) enrichment analysis using multiple R packages (e.g. clusterProfiler, enrichplot, ggplot2, and dplyr) (35–37).

### Development and verification of a novel ICD-based risk panel

#### Random assortment

The 128 OS samples in the GEO database were randomly stratified into two groups, including training dataset (n = 64) and test1 dataset (n = 64). All of 128 OS samples in the GEO database were assigned to the test2 cohort (n = 128) and all samples in the TARGET database were assigned to the test3 cohort (n = 84).

#### Risk panel development and validation

The univariate Cox regression analysis was carried out to determine DEGs that were highly related to prognosis in the training cohort. Second, to narrow the range of target genes, DEGs with prognostic values were included in least absolute shrinkage and seletion operator (LASSO) regression analysis. Subsequently, according to the result of multivariate Cox regression analysis, we developed a novel ICD-associated prognostic panel (ICD-APP) and calculated the risk score of it through the “predict” function in R. The median of the risk score was set as the cut-off point, and patients were stratified into high- and low-risk subpopulations. Kaplan–Meier survival curves were then depicted to analyze the survival discrepancy and receiver operating characteristic (ROC) curves of 1, 3 and 5 years were drawn to estimate the efficiency of our ICD-APP using the timeROC package. The above analysis was verified through the internal validation dataset (test1 and test2) and external validation dataset (test3). After identifying essential genes required for model construction, we queried the DisNor database (https://disnor.uniroma2.it/) to investigate these genes’ upstream and downstream connections and their mechanism of action (38, 39).

## Results

### Quality control, normalization, and bioinformatics analysis of scRNA-seq data

By filtering the single cell data such that each gene must be expressed in at least three cells and each cell must express at least 300 genes, a total of 49744 cells were gathered. Next, 28968 cells are obtained by calculating the proportions of mitochondria and rRNA using the PercentageFeatureSet function and ensuring that the expression of genes in each cell is between 300 and 5000, the content of mitochondria is less than 10 percent, and the UMI of each cell is at least greater than 1000. **Table 1** displays the cell count data for each sample before and after filtering. There is a strong association between the number of UMI and mRNA, as shown in **Figure S1A**, however there is no correlation between the number of UMI/mRNA and the content of mitochondrial genes. **Figures S1B, C** shows the violin before and after quality assurance. Principal component analysis (PCA) was used to estimate the available dimensions, and the findings did not indicate any substantial distinction between osteosarcoma cells. Fifty of the most distinctive principal components were chosen for further investigation (**Figure S1D**).

**Table 1:** Cell counts for each sample before and after filtering.

GSM	Patient	Sex	Age	raw_count	clean_count	percent %
GSM4952363	OS1	Male	16	8447	4763	56.39
GSM4952364	OS2	Female	19	8085	3905	48.30
GSM4952365	OS3	Female	45	9525	3550	37.27
GSM5155198	OS4	Male	19	4214	2696	63.98
GSM5155199	OS5	Male	14	10255	7701	75.10
GSM5155200	OS6	Male	13	9218	6353	68.92

**Figure 1:**
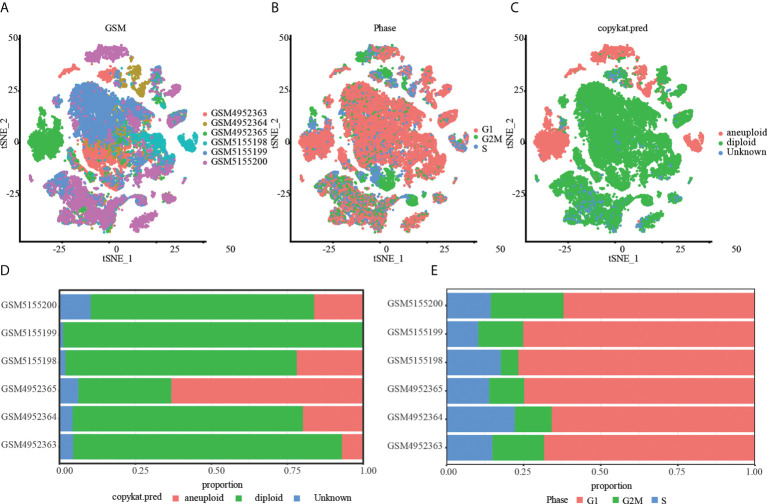
Tumor and normal cells of OS. **(A)** A t-SNE map of the distribution of cells in each OS sample, and each color represents the cells in each sample. **(B)** The t-SNE diagram shows the distribution traits of cells cycles marked with different colors. **(C)** The t-SNE diagrams of tumor and normal cells in OS samples are represented by different colors. **(D)** The proportion of tumor and normal cells in each OS sample. **(E)** Proportion of G1, G2/M and S phase cells in each OS sample.

Then, the RunTSNE function is used to assess the TSNE dimension reduction of 28968 cells, and **Figure 1A** represents the tsne diagram of the distribution of six samples. Using the marker gene in S phase and G2M phase, the CellCycleScoring function generated the cell cycle stage score, and **Figure 1B** displayed the distribution of cells in various cell cycles. In parallel, we examined single cell data *via* cnv using the copykat package. The results showed that there were 4988 tumor cells, 22589 normal cells and 1391 unknown cells (**Figure 1C**). Lastly, we evaluated the ratio of tumor cells to normal cells as well as the ratio of cells in the G1, G2M, and S phases in various samples. As depicted in **Figure 1D**, the percentage of normal cells in the majority of OS samples was much greater than that of malignant cells. In addition, the majority of cells are in the G1 phase, and the percentage of G2M phase cells is nearly equivalent to that of S phase cells (**Figure 1E**).

**Figure 2:**
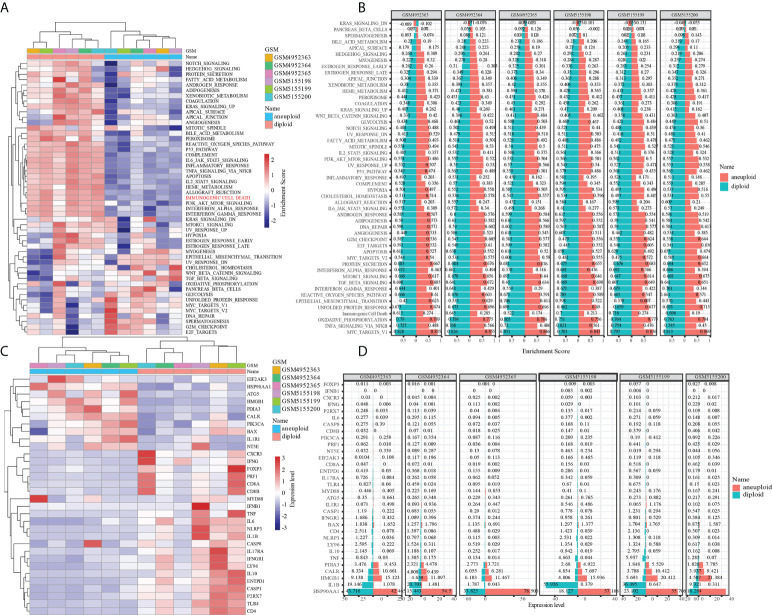
The role of immunogenic cell death in tumor and normal cells of OS. **(A)** Difference in activation of biological pathways between tumor and normal cells in OS. **(B)** Difference in expresion of ICD-related genes between tumor and normal cells in OS. **(C)** The enrichment scores of different signal pathways in normal and malignant cells of each osteosarcoma sample. **(D)** The expresion of ICD-related genes in normal and malignant cells of each osteosarcoma sample.

After distinguishing tumor cells from OS tissues, ssGSEA was applied to compute the enrichment scores of HALLMARK and ICD-associated pathways in single cell. Our findings revealed that ICD scores of tumor cells were considerably lower than those of normal cells, suggesting that tumor might protect themselves and survive through suppressing ICD-related processes (**Figures 2A, B**). ICD-targeted intervention might encourage tumor cell death and improve patients’ prognoses. Likewise, most ICD-related genes, including IL-6, IL-10, NLRP3, CD8A, CD8B, and TLR4, exhibited attenuated expression levels in tumor cells compared to normal cells (**Figures 2C, D**). Finally, we depicted the single-cell subpopulation distributions of ICD-related genes (**Figures S2** and **S3**)

### Identification of ICD-based molecular clusters in OS using consensus clustering analysis

**Figure 3:**
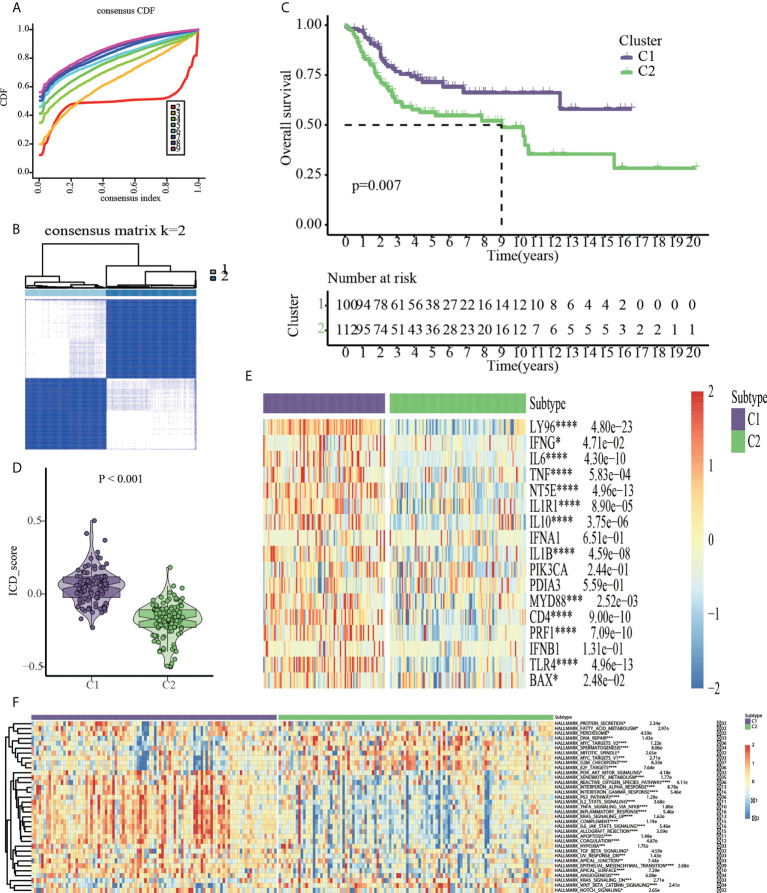
Consensus clustering based on the 17 ICD-related genes. **(A)** Cumulative distribution function (CDF). **(B)** 212 OS samples from GEO and TARGET platforms were divided into cluster1 (n=100) and cluster2 (n=112) by consensus clustering. **(C)** Kaplan-Meier analysis indicated cluster1 had more favorable prognosis than cluster2. **(D)** ICD scores based on ssGSEA algorithm. **(E)** Cluster heatmap of 17 ICD-related genes. **(F)** HALLMARK pathway activities based on ssGSEA algorithm *p < 0.05, **p < 0.01, ***p < 0.001, ****p < 0.0001.

To discover ICD-based molecular clusters of OS, consensus clustering analysis was performed using 17 ICD-related genes. In consideration of cumulative distribution function (CDF) curves and Delta area, k = 2 was selected as the number of unique and nonoverlapping subtypes (**Figures 3A, B**). Thus, two ICD-based molecular clusters of OS were constructed, with cluster 1 including 100 cases and cluster 2 containing 112 cases. Two ICD-related groups revealed statistically distinct survival curves (**Figure 3C**). Patients with OS in cluster 1 had a survival advantage and higher ICD scores than those in cluster 2, indicating the protective significance of ICD in OS patients (**Figure 3C, D**). Significantly different ICD-related gene expression levels were observed between the C1 and C2 subtypes, with the majority of genes being overexpressed in the C1 subtype (**Figure 3E**). The findings of the pathway-based ssGSEA demonstrated that the C1 subtype activates a greater number of tumor-related pathways, such as PI3K_AKT_MTOR_SIGNALING, INTERFERON_ RESPONSE, P53_PATHWAY, INFLAMMATORY_RESPONSE, KRAS_SIGNALING, APOPTOSIS, HYPOXIA, TGF_BETA_SIGNALING, and EPITHELIAL_MESENCHYMAL_TRANSITION, suggesting the close association of ICD with above typical tumor-associated pathways (**Figure 3F**).

### Cluster-based analysis of drug sensitivity

**Figure 4:**
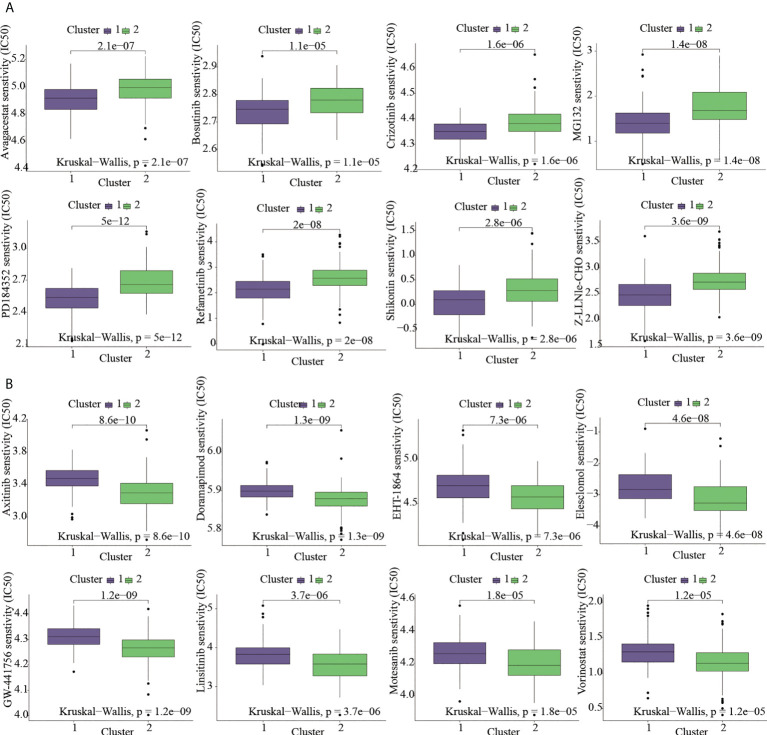
Targeted-drug sensitivity prediction. **(A)** The drugs favoring C1 subtype. **(B)** The drugs favoring C2 subtype.

Given that molecularly targeted medicines are currently widely used to treat OS, the chemotherapeutic response of ICD-based clusters was systematically evaluated using the “pRRophetic” package in R. Our data showed that Avagacestat, Bosutinib, Crizotinib, MG132, PD184352, Refametinib, Shikonin, Z-LLNle-CHO were expected to benefit C1 subtype; however, C2 subtype was more benefical from Axitinib, Doramapimod, EHT-1864, Elesclomol, GW-441756, Linsitinib, Motesanib, Vorinostat (**Figures 4A, B**).

### Cluster-based analysis of tumor immune microenvironment

**Figure 5:**
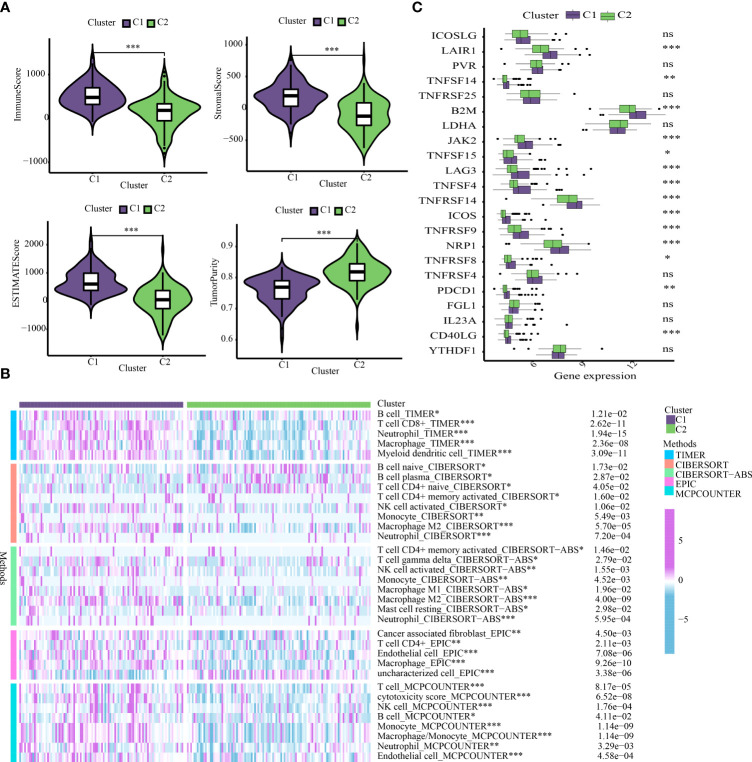
Cluster-based analysis of tumor immune microenvironment. **(A)** Comparison of tumor immune microenvironment components. **(B)** The distribution traits of immunocyte infiltration in ICD-based clusters. **(C)** The discrepancy in expression levels of immune checkpoints. *p < 0.05, **p < 0.01, ***p < 0.001, ns, not statistically significant.

The “ESTIMATE” R package was utilized to assess the discrepancy in the immune characteristics between C1 and C2 subtypes (predicated on the StromalScore, ImmuneScore, ESTIMATEScore, and Tumorpurity) utilizing the transcriptome data. Our results revealed that C1 subtype exhibited enhanced levels of immuneScore, stromalScore, and estimateScore, but showed attenuated levels of tumor purity (**Figure 5A**). These results indicated that OS prognosis positively correlated to Immune and Stromal components. To further explore the abundance of immunocyte-infiltrating in the tumor microenvironment, a variety of algorithms were applied to estimate the percentage of the immune cell infiltrate in C1 and C2 subtypes. As depicted in **Figure 5B**, C1 subtype showed an enhanced proportion of B cells, CD4+ T cells, CD8+ T cells, macrophages, neutrophils, NK cells and myeloid dendritic cells based on TIMER, CIBERSORT-ABS, EPIC and MCPCOUNTER algorithms. It has been well-established that B cells are usually divided into four lineages-naive B cells, activated B cells, effector B cells (i.e. plasma cells), and memory B cells. Among them, naive B cells and plasma cells have a higher proportion in the C2 subtype based on CIBERSORT algorithm. ICGs are determining factors towards immune cells to perform immune function. Likewise, our findings revealed that C2 subtype exhibited enhanced expression of ICGs (**Figure 5C**).

### Cluster-based analysis of DEGs

A total of 212 DEGs were obtained depending on the thresholds in the methods section (**Table S1**). The DEGs-based GO enrichment analysis demonstrated that 699 biological process (BP), 36 cellular component (CC), and 53 molecular function (MF) terms had the significant differences between the two subtypes (FDR < 0.05). The first 18 GO terms were depicted in **Figure S4A**. The obviously enriched pathways were revealed by the KEGG pathway analysis of DEGs (FDR < 0.05). Further visualization of the top 18 enriched pathways demonstrated that genes were considerably enriched in tumor-related pathways such as the NF-κB signaling pathway, Toll-like receptor signaling pathway, complement and coagulation cascades, cytokine-cytokine receptor interaction (**Figure S4B**).

#### Risk model development of OS based on ICD-related genes

The DEGs-based univariate Cox analysis between the C1 and C2 subtypes identified 12 prognostic genes in the train cohort (**Table S2**). Lasso regression analysis was performed to further exclude the unnecessary prognostic genes. **Figure S5A** showed the locus of each independent variable. The number of independent variables tending to zero also enhanced accompany with the increase of lambda (λ) value (**Figure S5B**). The pattern was constructed by performing a10-fold cross-validation, and **Figure S5C** showed the confidence interval under each λ. Subsequently, a total of six ICD-related genes (i.e., BAMBI, TMCC2, NOX4, DKK1, POPDC3, and CBS) were preserved for further multivariate Cox regression analysis. Ultimately, a novel ICD-APP was constructed by integrating five-gene expressions (i.e., BAMBI, TMCC2, NOX4, DKK1, and CBS). The DisNor database was then utilized to identify the upstream and downstream genes reacted with BAMBI, TMCC2, NOX4, DKK1, and CBS (**Figure S5D**). FZD5 and DVL2 are neighbouring downstream of BAMBI. E2F1 acts to promote the formation of superoxide and reactive oxygen species following activation of NOX4 (**Figure S5D**). KREMEN1 and KREMEN2 are neighbouring downstream of DKK1. USF1, NFYA, SP3, and SP1 are neighbouring upstream of CBS (**Figure S5D**).

**Figure 6:**
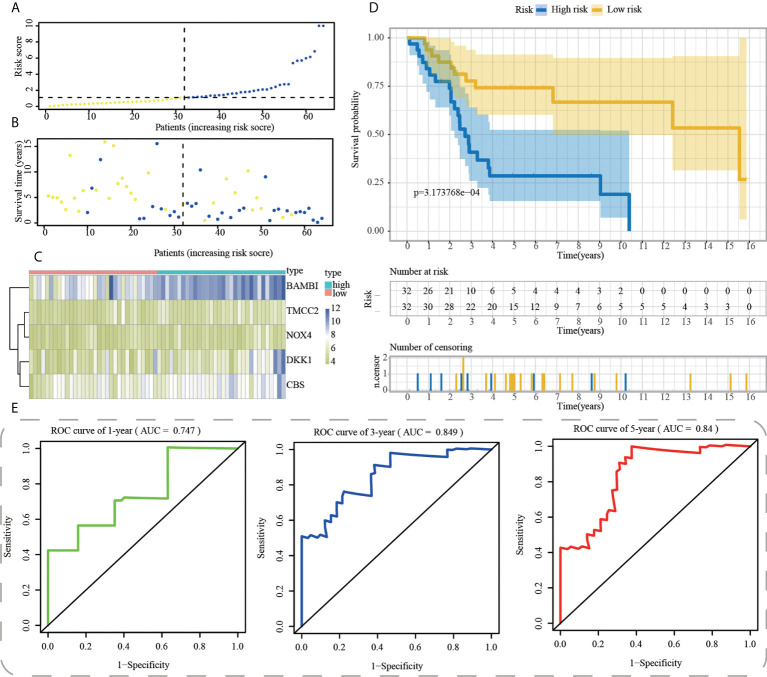
Construction of ICD-APP in the training cohort. **(A)** Discriminate high- and low-subpopulations in the training cohort. **(B)** The relationship of survival status and risk score in the training cohort. **(C)** The distribution traits of the expression of 5 genes used for model development in the training cohort. Evaluate the prognostic performances of ICD-APP in the training cohort **(D)** KM survival curves; **(E)** time-dependent ROC curves.

Afterwards, we used R’s “predict” function to get the median risk score across all samples and then used it to categorise them into high- and low-risk subgroups (**Figure 6A**). **Figure 6B** depicts a higher mortality rate in the high-risk category of PAAD patients based on the distributions of risk scores and survival status. The heatmap depicted the expressions of mRNA and lncRNAs in the prognostic signature (**Figure 6C**). Consistently, survival analyses with the Kaplan-Meier method showed that OS patients had a worse clinical outcome in high-risk subgroup, suggesting that the prognosis of OS patients with different risk stratification could be accurately distinguished by our ICD-APP (**Figure 6D**). We further verified the effectiveness and accuracy of our ICD-APP. Receiver operating characteristic (ROC) analysis was used to determine the diagnostic performance of the risk score. The 1-year, 3-year, and 5-year area under the curve (AUC) values of the risk score were 0.747, 0.849, and 0.840, respectively (**Figure 6E**).

#### Internal and external verification of the ICD-APP in OS

**Figure 7:**
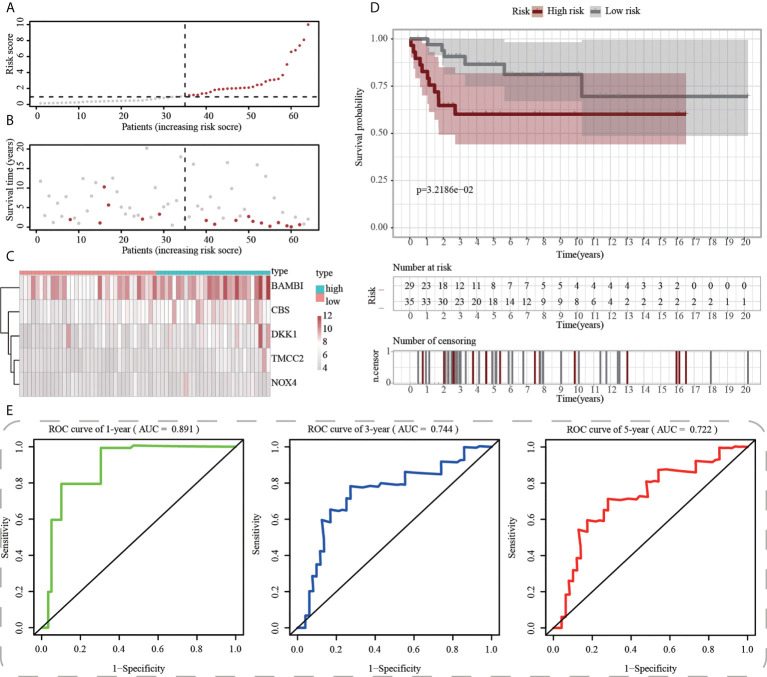
Internal validation of ICD-APP in the test1 cohort. **(A)** Discriminate high- and low-subpopulations in the test1 cohort. **(B)** The relationship of survival status and risk score in the test1 cohort. **(C)** The distribution traits of the expression of 5 genes used for model development in the test1 cohort. Evaluate the prognostic performances of ICD-APP in the test1 cohort **(D)** KM survival curves; **(E)** time-dependent ROC curves.

**Figure 8:**
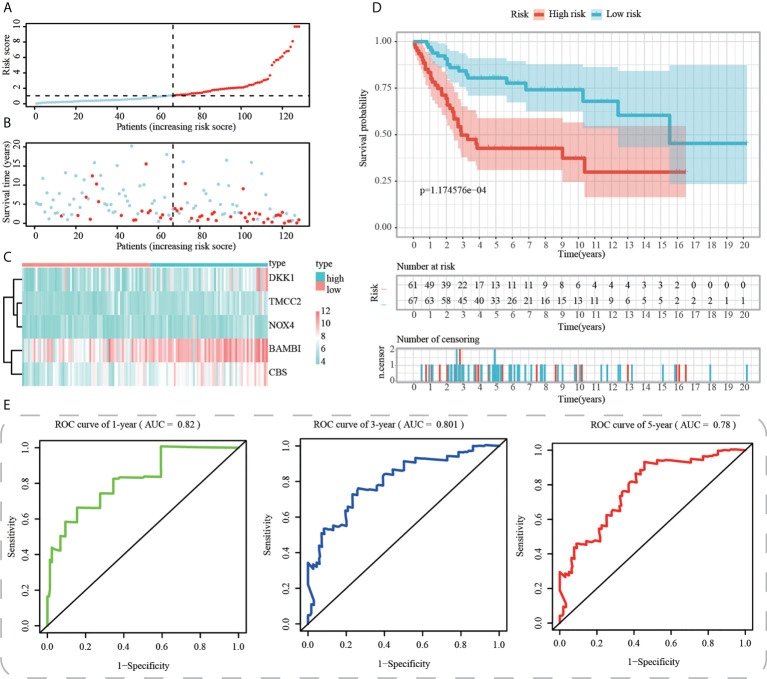
Internal validation of ICD-APP in the test2 cohort. **(A)** Discriminate high- and low-subpopulations in the test2 cohort. **(B)** The relationship of survival status and risk score in the test2 cohort. **(C)** The distribution traits of the expression of 5 genes used for model development in the test2 cohort. Evaluate the prognostic performances of ICD-APP in the test2 cohort **(D)** KM survival curves; **(E)** time-dependent ROC curves.

**Figure 9:**
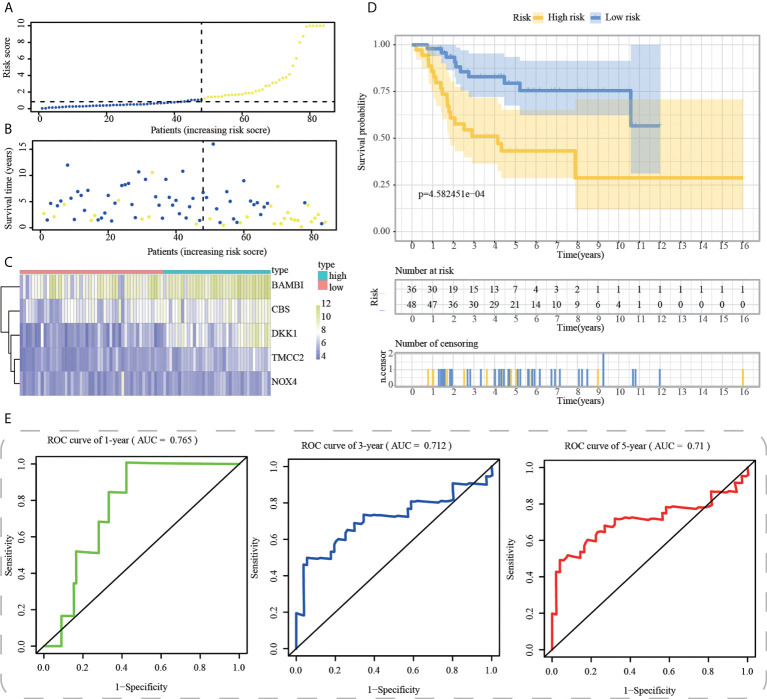
External validation of ICD-APP in the test3 cohort. **(A)** Discriminate high- and low-subpopulations in the test3 cohort. **(B)** The relationship of survival status and risk score in the test3 cohort. **(C)** The distribution traits of the expression of 5 genes used for model development in the test3 cohort. Evaluate the prognostic performances of ICD-APP in the test3 cohort **(D)** KM survival curves; **(E)** time-dependent ROC curves.

First, using the median risk score in the train cohort as the standard, patients from tests 1, 2, and 3 were divided into high-risk and low-risk subpopulations, respectively (**Figures 7A**-**9A**). The distributions of survival status and risk scores were comparable in the internal validation (test 1 and test 2 cohorts) and external validation (test 3 cohort) compared to the training cohort (**Figures 7B**-**9B**). In both internal and external verification cohorts, heatmaps obtained from three test cohorts revealed the presence of genes with high expression (BAMBI, TMCC2, NOX4, DKK1, and CBS) in the high-risk group (**Figures 7C**-**9C**). Furthermore, patients with high-risk scores had worse unfavorable overall survival rates in the test1 test2 and test3 groups (**Figures 7D**-**9D**). As for the diagnostic value of ICD-APP, the AUC values of the ROC curves were 0.891, 0.744, and 0.722 in the test1 cohort, 0.820, 0.801, and 0.780 in the test2 cohort, and 0.765, 0.712, and 0.710 in the test3 cohort for 1-, 3-, and 5-year survival, respectively (**Figures 7E**-**9E**). Overall, test1 and test2 findings were compatible with train cohort results, and test3 results were consistent with both internal and external verifiers.

## Discussion

With a high propensity for invasion and metastasis, OS is the most prevalent malignant bone tumor in both adults and children. At present, a large number of therapeutic projects have been applied for OS patients, which includes surgery, radiotherapy, chemotherapy, and neoadjuvant chemotherapy (40). However, the overall survival of OS patients still has a large gap to satisfaction, particularly for the advanced OS, due to its high malignancy (41). The malignant progression of OS commonly develops along with the expression changes of multiple genes, which may influence the prognosis of patients with OS (42). These genes were deemed as potential therapeutic targets for personalized treatment in tumor patients. Recently, along with the sequencing technology was rapidly developed, high-throughput genomics has been used for the exploration of tumor generation and progression-related genes (43, 44). Moreover, the deep investigation of the molecular mechanisms of tumorigenesis and development can be implemented by high-throughput genomics.

In this study, combination analysis of scRNA-seq and bulk RNA-seq data was performed to highlight the significant contributions of ICD in OS. After quality control and normalization of scRNA-seq data, all single cells were divided into two subpopulations (aneuploid and diploid cells). Significant down-regulation of ICD scores and ICD-related gene expression was detected in aneuploid cells compared to diploid cells. In addition to typical cell death (e.g. apoptosis and pyroptosis), a variety of chemo- and radiotherapy-strategies were recently reported to induce a new cell death process (i.e. ICD) and then improve the prognosis. Similarly, our findings indicated that compared to diploid cells, aneuploid cells might survive through inhibition of ICD activities. These results showed that ICD played a protective role in OS, and tumor cell might protect themselves through down-regulation of ICD activities.

After illustrating the protective roles of ICD in OS, we performed clustering analysis and risk stratification to distinguish OS patients with distinct ICD activities. First, 212 OS samples were genotyped based on the expression profiles of ICD-related genes, and two subtypes (C1 and C2) were obtained. The C1 subtype with a higher ICD score and favorable prognosis was more associated with many star pathways, such as PI3K/Akt/mTOR pathway, P53 pathway, KRAS signaling, inflammatory response, apoptosis, hypoxia, and TGF-β signaling. Tang et al. (45) reported that CXCR3 had the potential to modulate above signaling pathways of OS patients, recruited more immune infiltration of CD8+T cells, M1 macrophages, plasma cells, and activated NK cells, and then improved OS patients’ prognoses. These findings were in agreement that C1 subtype with a higher ICD score had a better clinical outcome, which was also verified the protective role of ICD in OS.

Considering the remarkable significance of targeted-drug therapy in OS, we intensively explored the discrepancy in drug sensitivity between C1 and C2. Notably, C1 subtype might be beneficial from Avagacestat, Bosutinib, Crizotinib, MG132, PD184352, Refametinib, Shikonin, and Z-LLNle-CHO, whereas, C2 subtype was more benefical from Axitinib, Doramapimod, EHT-1864, Elesclomol, GW-441756, Linsitinib, Motesanib, and Vorinostat. Overall, these findings might provide new insight for individual management of patients with OS.

To explore the underlying prognostic mechanism of the proposed ICD-based molecular subtype and investigate the reason that leads to prognostic differences among the different subtypes, we compared the tumor immune microenvironment among the different subtypes. Interestingly, C1 subtype with higher ICD scores and favorable clinical outcomes had significantly higher proportions of immunocyte infiltration (e.g. B cells, CD4+ T cells, CD8+ T cells, macrophages, neutrophils, NK cells and myeloid dendritic cells) and lower expression levels of immune checkpoints. CD8+ T cells are the essential effector cells against tumors, and the tumor-related antigens of Major Histocompatibility Complex I (MHC I) are recognized by activated CD8+ T cells, which then destroy tumor cells by activating their T cell receptors (46). As the primary effectors in humoral immunity, B cells can stimulate the T-cell response *via* producing immunoglobulin and prevent the tumor progressions by destroying tumor cells directly (47). Therefore, the high infiltration of immune cells is closely correlated to a favorable prognosis of OS.

A total of 212 DEGs between the C1 and C2 subtypes were identified using the limma package. We constructed a 5-gene signature based on the DKK1, TMCC2, NOX4, BAMBI, and CBS genes rooting in the 212 identified DEGs. It has been reported that DKK1 functioned as a prognostic or diagnostic marker for OS assessment, and DKK1 immunodepletion may also be used as an additional treatment for OS (48). TMCC2 acts as a member of transmembrane and coiled-coil domain family, and its-encoding proteins might locate in endoplasmic reticulum and invovled in amyloid precursor protein metabolic process and bone marrow hematopoiesis (49, 50). In addition, TMCC2 is identified as a potential biomarker of steroid-induced osteonecrosis of the femoral head (51). NOX4 may function as an oncogene in a variety of tumors, such as pancreatic cancer, breast cancer, and lung cancer; however, its potential role in OS has not been reported before (52–54). The overexpression of BAMBI promotes the growth and invasion of human osteosarcoma cells (55). CBS has a significant correlation to the OS prognosis and is evidenced as an independent prognostic factor (56). The comprehensive effect of ICD regulated by these genes in OS was reported for the first time in this study.

The Kaplan–Meier survival analysis indicated that the risk score could clearly distinguished OS patients with a favourable or unfavourable prognosis, and the time-dependent ROC curves demonstrated the risk score’s high accuracy in predicting the clinical outcomes of OS in both internal validation and external validation cohorts.

However, despite these advances, there remain certain limitations. First, as a retrospective study of OS, our study lacked of clinical prospective studies, which should be performed for the verification of the prognostic characteristic and the stability of the 5-gene prognostic model. Additionally, the molecular mechanisms by which DKK1, TMCC2, NOX4, BAMBI, and CBS promote the malignant progression of OS still require a deeper investigation.

## Conclusions

Taken together, the ICD-based molecular classifier of OS was identified, and a 5- gene prognostic signature was developed to predict prognostic risk in patients with OS.

## Data availability statement

The datasets presented in this study can be found in online repositories. The names of the repository/repositories and accession number(s) can be found in the article/**Supplementary Material**.

## Author contributions

JiY, JZ, and SN contributed to this study equally. All the authors participated in the design of study, data collection and processing, bioinformatics analysis, and writing and revising the manusript. All authors read and approved the final manuscript. All authors contributed to the article and approved the submitted version.

## Funding

This study was supported by National Natural Science Foundation of China (No.82002908).

## Acknowledgments

We thank Bullet Edits Limited for the linguistic editing of the manuscript. We also thank Dr. Sida Liu and Mr. Qihang Yuan for their selfless help and guidance.

## Conflict of interest

The authors declare that the research was conducted in the absence of any commercial or financial relationships that could be construed as a potential conflict of interest.

## Publisher’s note

All claims expressed in this article are solely those of the authors and do not necessarily represent those of their affiliated organizations, or those of the publisher, the editors and the reviewers. Any product that may be evaluated in this article, or claim that may be made by its manufacturer, is not guaranteed or endorsed by the publisher.

## Supplementary material

Supplementary Figure 1 scRNA-seq analysis of 6 osteosarcoma samples. **(A)** The correlation between mitochondrial gene and the number of UMI/mRNA, and the relationship between the number of UMI and mRNA. **(B, C)** Quality control, including the number of unique genes and total molecules, the percentage of reads that map to the mitochondrial genome. **(D)** The PCA based on scRNA-seq data confirms top 50 PCs.

Supplementary Figure 2–3 Expression distributions of ICD-related genes in single-cell levels.

Supplementary Figure 4 Functional annotation of DEGs between C1 and C2 subtype. **(A)** GO functional enrichment analysis of DEGs. **(B)** KEGG functional enrichment analysis of DEGs.

Supplementary Figure 5 Variable selection and prediciton of upstream and downstream genes. **(A-C)** Five genes selected by LASSO-Cox regression analysis. **(D)** The network of BAMBI, TMCC2, NOX4, DKK1, and CBS in DisNor database.
