# Supplementary figures and images for: Integration of single-cell RNA sequencing and bulk RNA sequencing to reveal an immunogenic cell death-related 5-gene panel as a prognostic model for osteosarcoma
Source: Front Immunol. 2022 Sep 26;13:994034. doi: 10.3389/fimmu.2022.994034 (PMC9549151; doi:10.3389/fimmu.2022.994034)

**CASP1**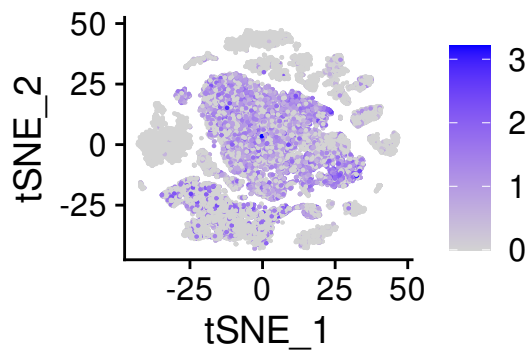**IL1R1**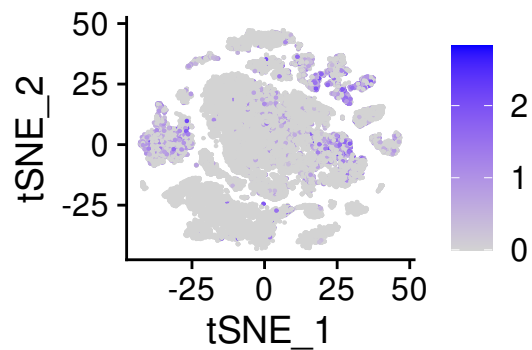**IL1B**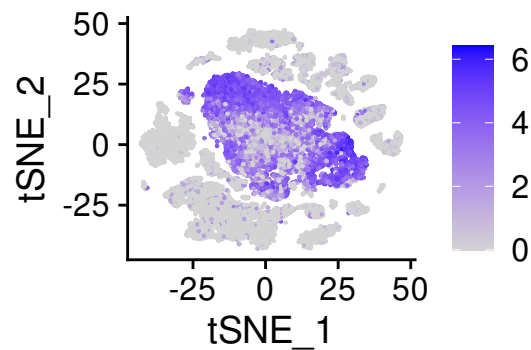**NLRP3**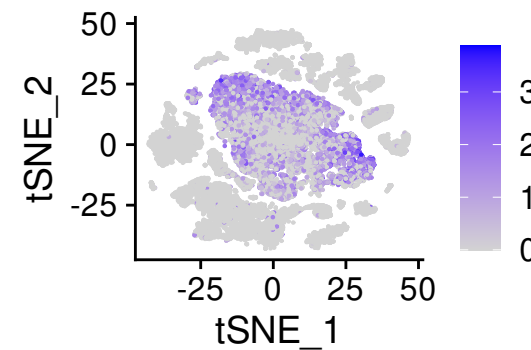**P2RX7**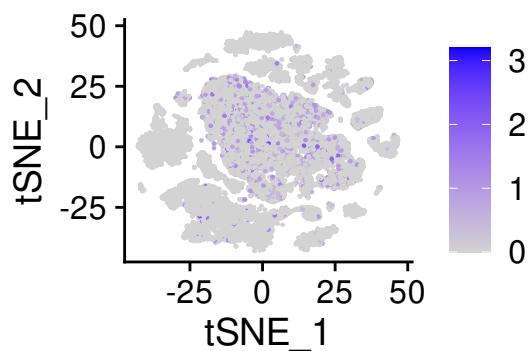**LY96**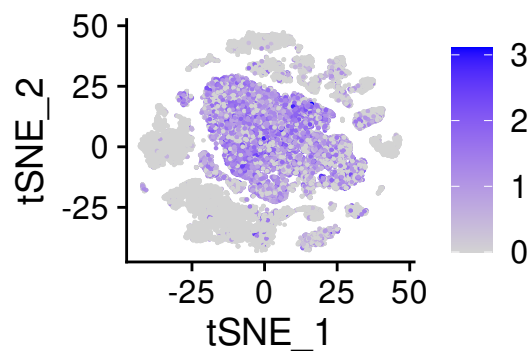**MYD88**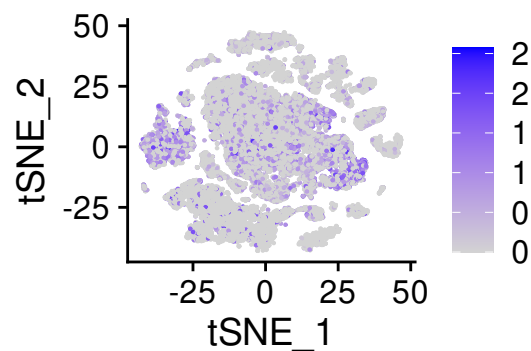**TLR4**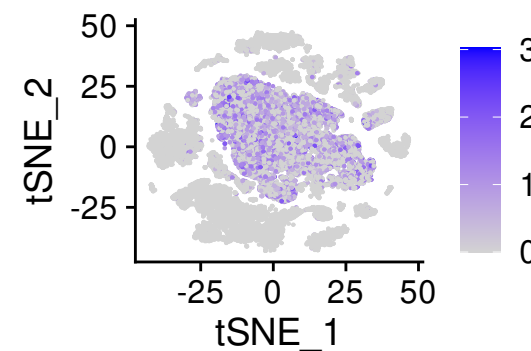**CD4**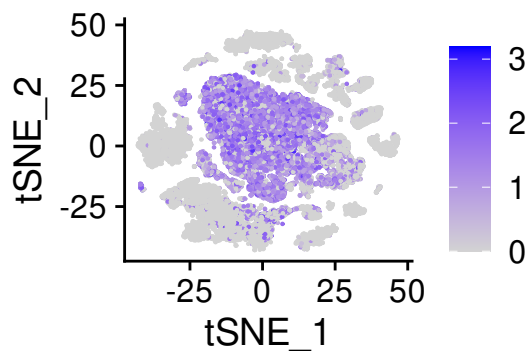**CD8B**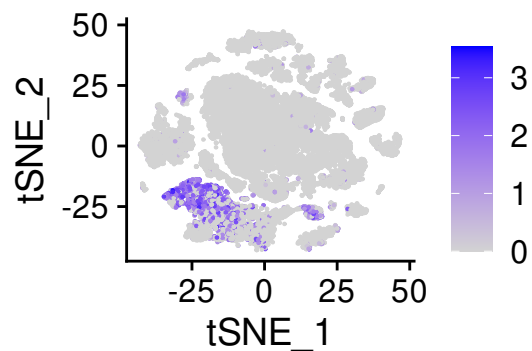**CD8A**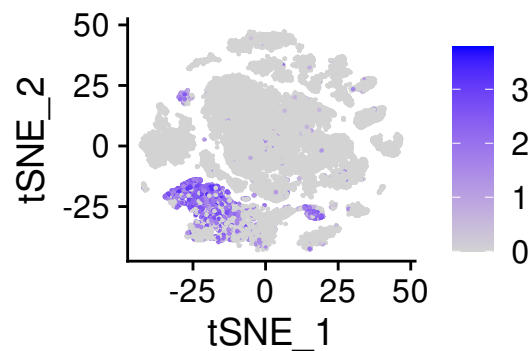**FOXP3**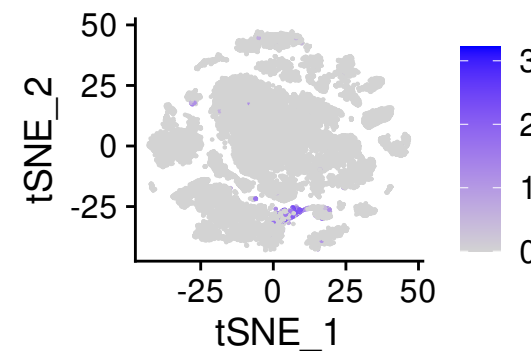**IFNG**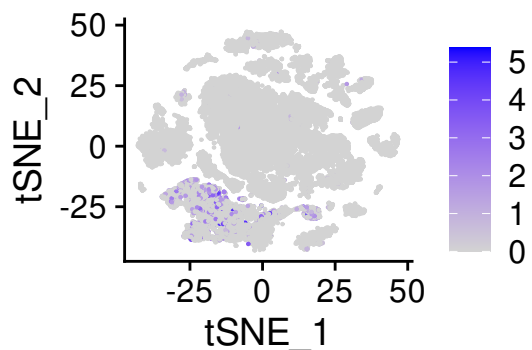**IFNGR1**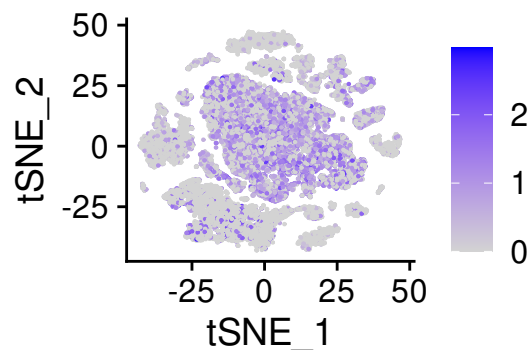**IL17RA**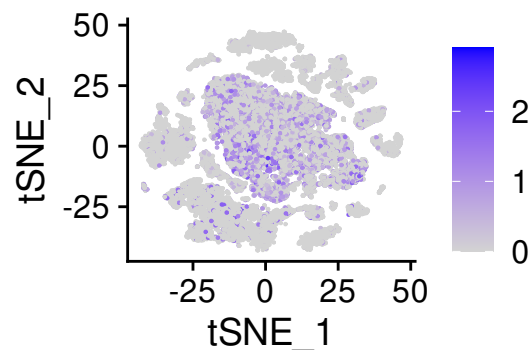**PRF1**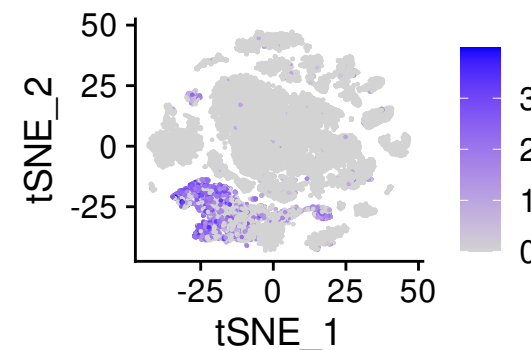

Supplement: Supplementary file 2 [file DataSheet_3.pdf]

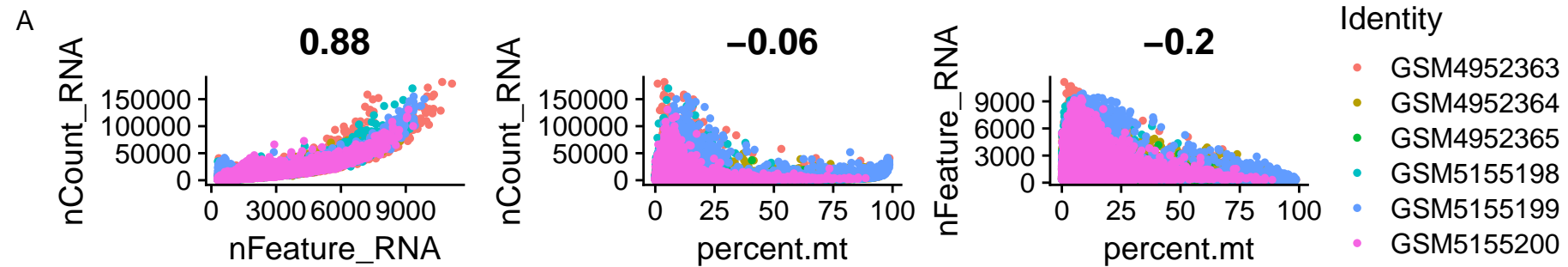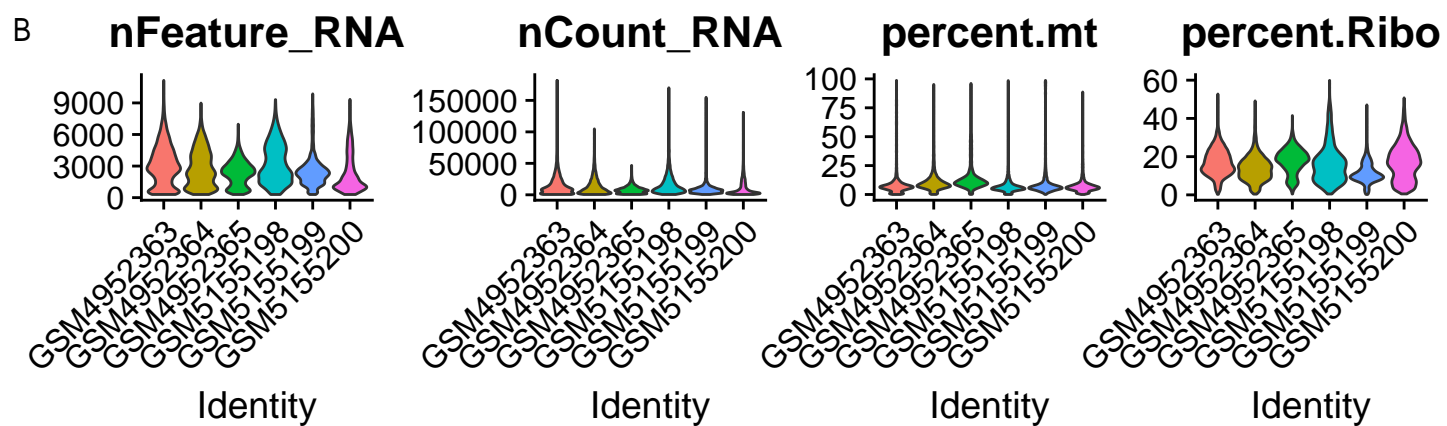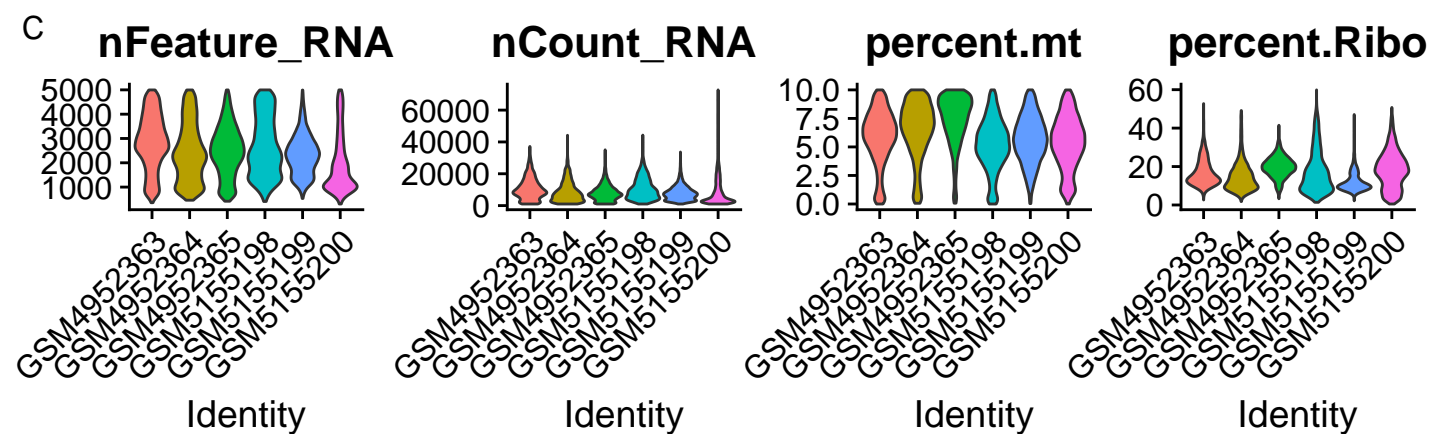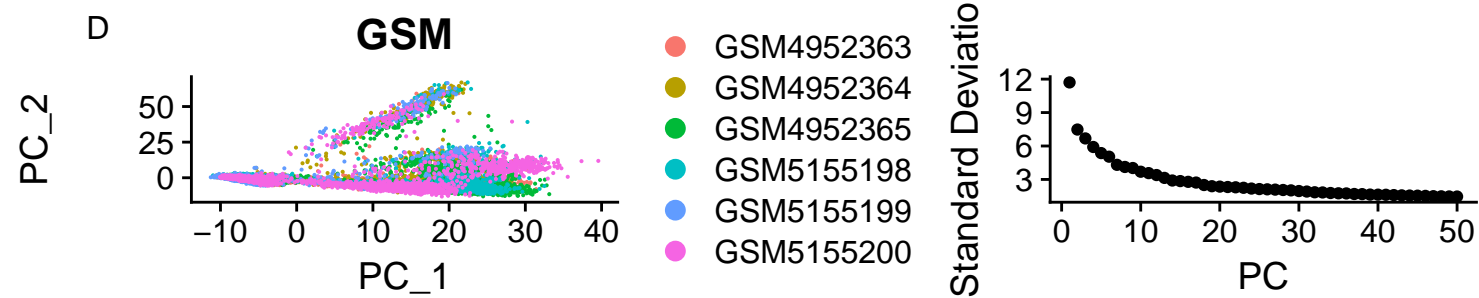

Supplement: Supplementary Figure 1 — scRNA-seq analysis of 6 osteosarcoma samples. (A) The correlation between mitochondrial gene and the number of UMI/mRNA, and the relationship between the number of UMI and mRNA. (B, C) Quality control, including the number of unique genes and total molecules, the percentage of reads that map to the mitochondrial genome. (D) The PCA based on scRNA-seq data confirms top 50 PCs. [file DataSheet_1.pdf]

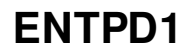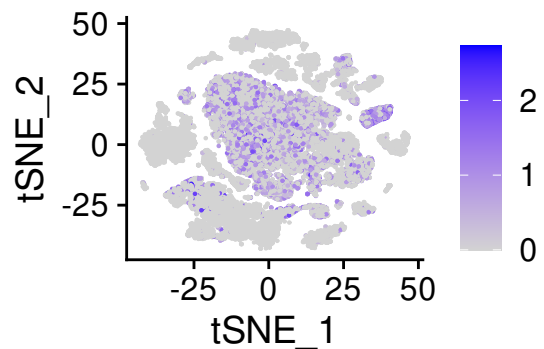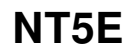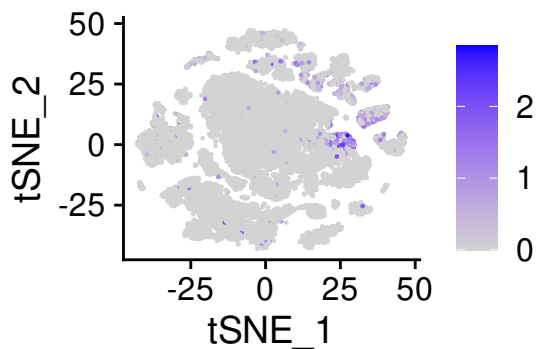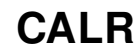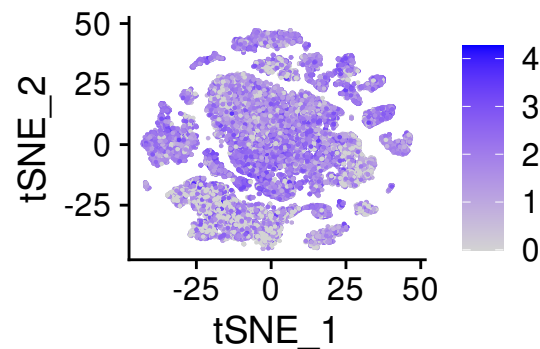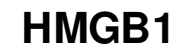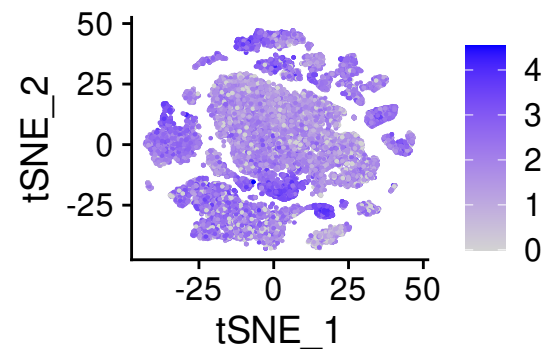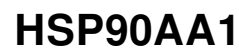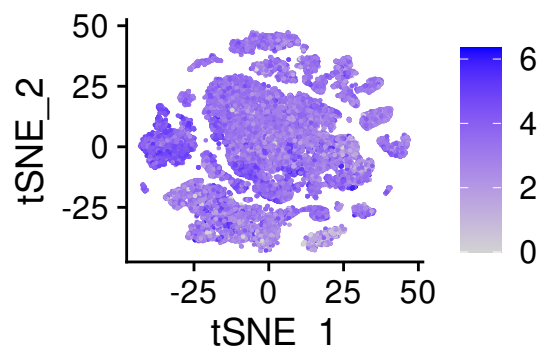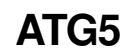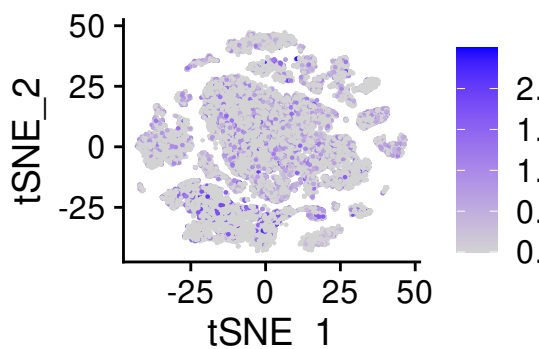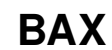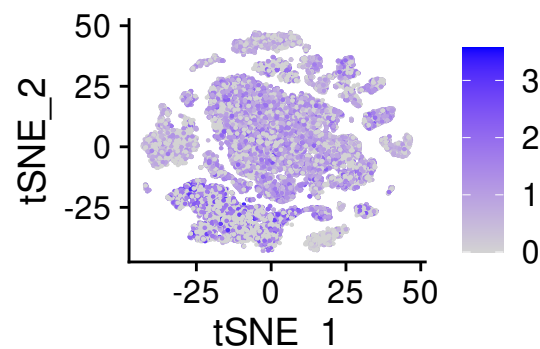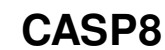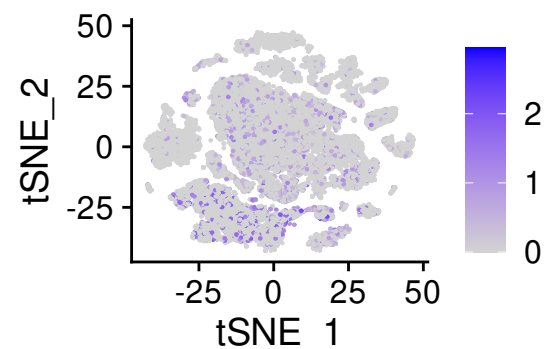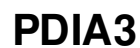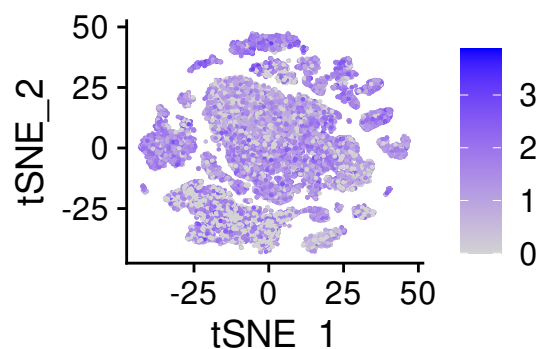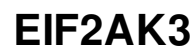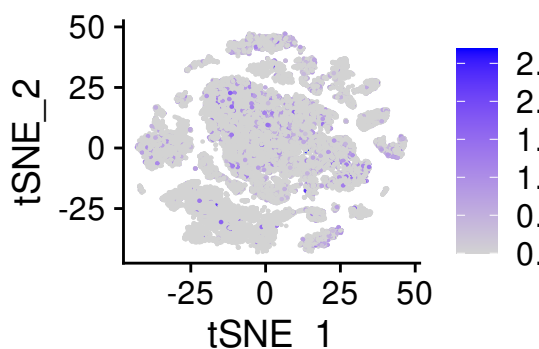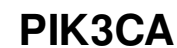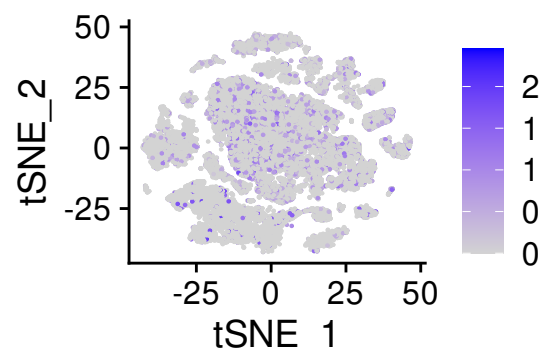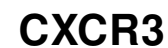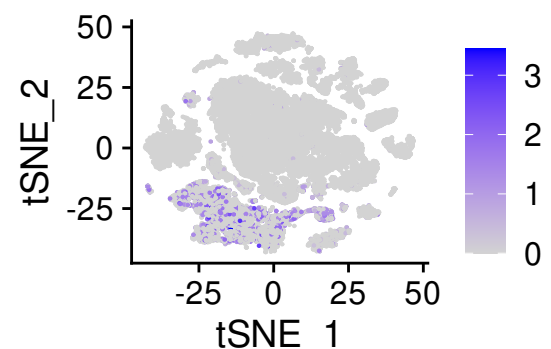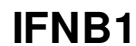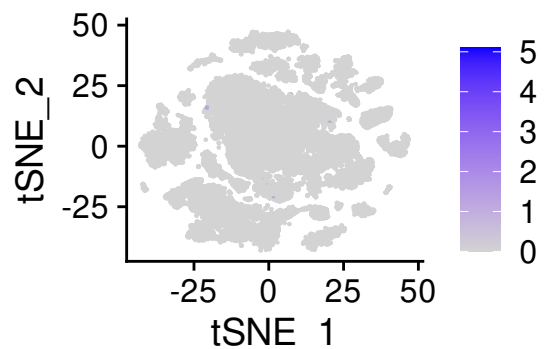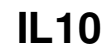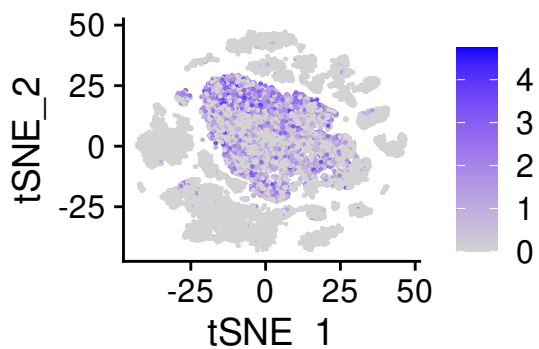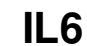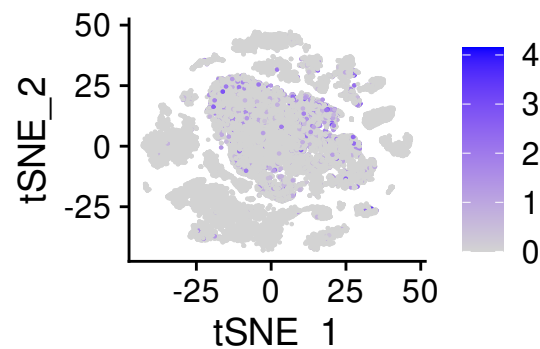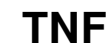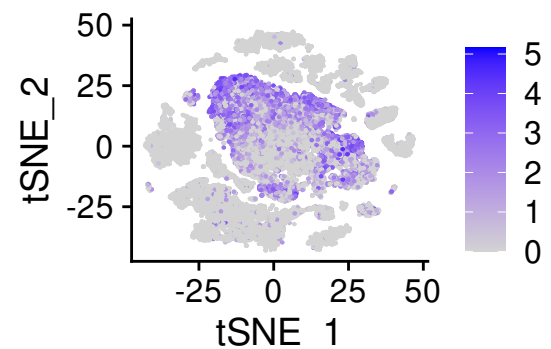

Supplement: Supplementary Figure 2–3 — Expression distributions of ICD-related genes in single-cell levels. [file DataSheet_2.pdf]
