# Supplementary material for: Integration of single-cell RNA sequencing and bulk RNA sequencing to reveal an immunogenic cell death-related 5-gene panel as a prognostic model for osteosarcoma
Source: Front Immunol. 2022 Sep 26;13:994034. doi: 10.3389/fimmu.2022.994034 (PMC9549151; doi:10.3389/fimmu.2022.994034)
Supplement: Supplementary Figure 4 — Functional annotation of DEGs between C1 and C2 subtype. (A) GO functional enrichment analysis of DEGs. (B) KEGG functional enrichment analysis of DEGs. [file DataSheet_4.pdf]

A B

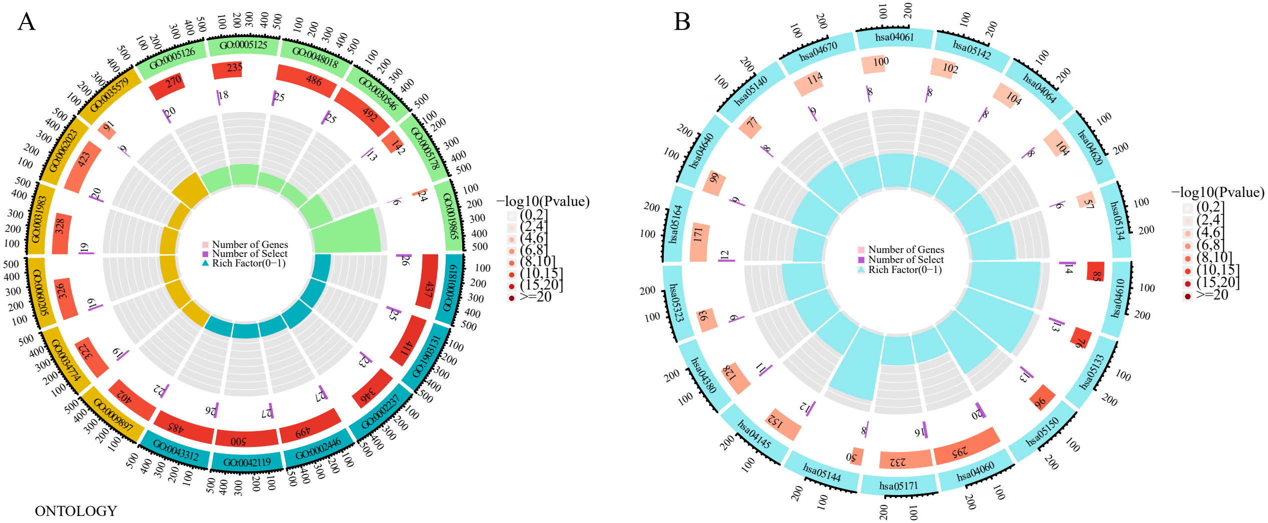

ONTOLOGY

Biological Process

GO： 0001819 ： positive regulation of cytokine production
GO： 1903131 ： mononuclear cell differentiation
GO： 0002237 ： response to molecule of bacterial origin
GO： 0002446 ： neutrophil mediated immunity
GO： 0042119 ： neutrophil activation
GO： 0043312 ： neutrophil degranulation

Cellular Component

GO： 0009897 ： external side of plasma membrane
GO： 0034774 ： secretory granule lumen
GO： 0060205 ： cytoplasmic vesicle lumen
GO： 0031983 ： vesicle lumen
GO： 0062023 ： collagen-containing extracellular matrix
GO： 0035579 ： specific granule membrane

Molecular Function

GO： 0005126 ： cytokine receptor binding
GO： 0005125 ： cytokine activity
GO： 0048018 ： receptor ligand activity
GO： 0030546 ： signaling receptor activator activity
GO： 0005178 ： integrin binding
GO： 0019865 ： immunoglobulin binding

Pathway

KEGG

Hsa04061:Viral protein interaction with cytokine and cytokine receptor
Hsa05142:Chagas disease
Hsa040664:NF-kappa B signaling pathway
Hsa04620:Toll-like receptor signaling pathway
Hsa05134:Legionellosis
Hsa04610:Complement and coagulation cascades
Hsa05133:Pertussis
Hsa05150:Staphylococcus aureus infection
Hsa04060:Cytokine-cytokine receptor interaction
Hsa05171:Coronavirus disease - COVID-19
Hsa05144:Malaria
Hsa04145:Phagosome
Hsa04380:Osteoclast differentiation
Hsa05323:Rheumatoid arthritis
Hsa05164:Influenza A
Hsa04640:Hematopoietic cell lineage
Hsa05140:Leishmaniasis
Hsa04670:Leukocyte transendothelial migration
