# Supplementary material for: Integration of single-cell RNA sequencing and bulk RNA sequencing to reveal an immunogenic cell death-related 5-gene panel as a prognostic model for osteosarcoma
Source: Front Immunol. 2022 Sep 26;13:994034. doi: 10.3389/fimmu.2022.994034 (PMC9549151; doi:10.3389/fimmu.2022.994034)
Supplement: Supplementary Figure 5 — Variable selection and prediciton of upstream and downstream genes. (A-C) Five genes selected by LASSO-Cox regression analysis. (D) The network of BAMBI, TMCC2, NOX4, DKK1, and CBS in DisNor database. [file DataSheet_5.pdf]

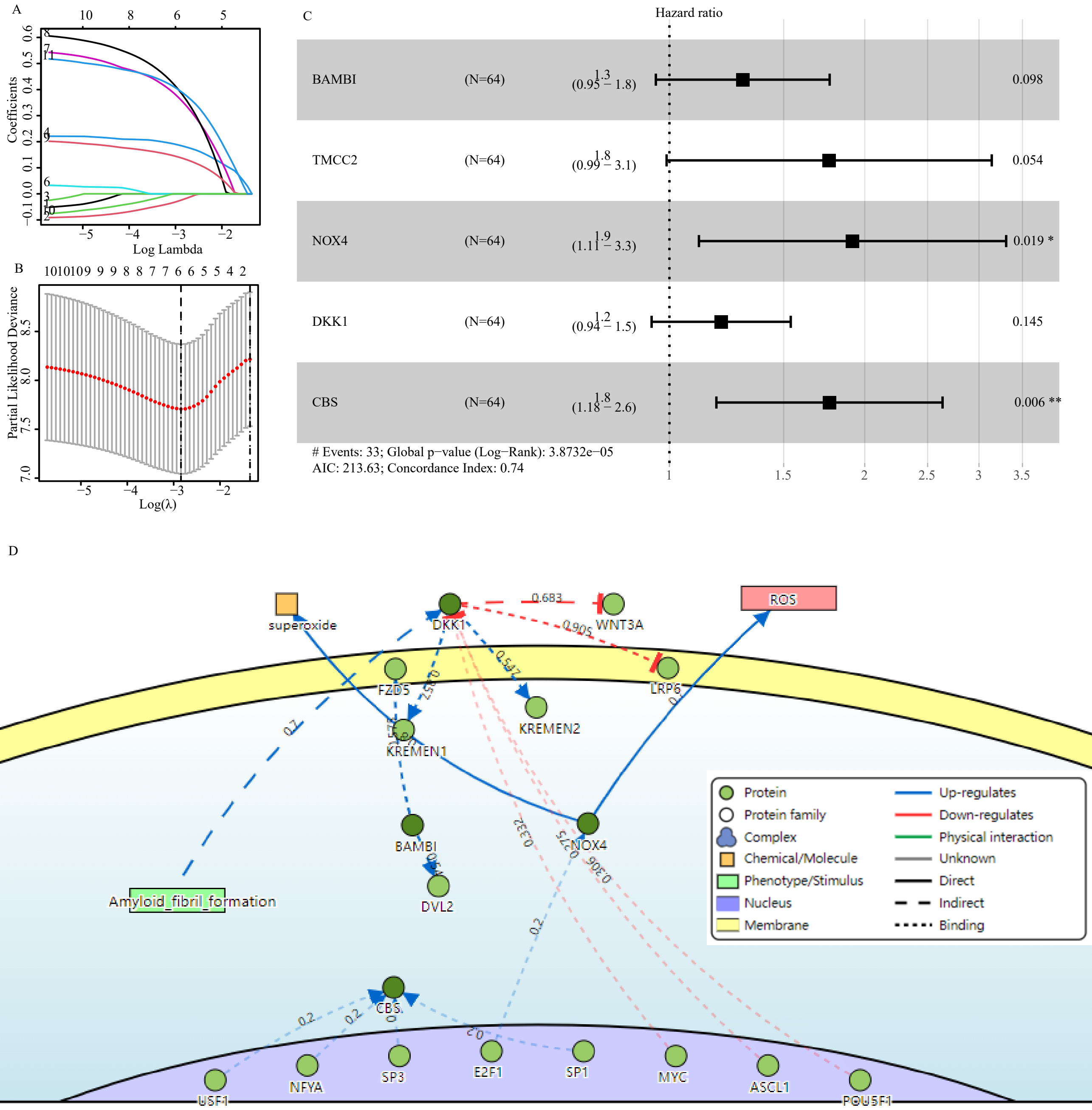

A
Coefficients
Log Lambda
B
Partial Likelihood Deviance
Log(λ)
C
Hazard ratio
BAMBI (N=64) 1.3 (0.95 – 1.8) 0.098
TMCC2 (N=64) 1.8 (0.99 – 3.1) 0.054
NOX4 (N=64) 1.9 (1.11 – 3.3) 0.019 *
DKK1 (N=64) 1.2 (0.94 – 1.5) 0.145
CBS (N=64) 1.8 (1.18 – 2.6) 0.006 **
Events: 33; Global p−value (Log−Rank): 3.8732e−05
AIC: 213.63; Concordance Index: 0.74
D
superoxide
DKK1
WNT3A
ROS
FZD5
LRP6
KREMEN2
KREMEN1
BAMBI
NOX4
DVL2
Amyloid_fibril_formation
CBS
USF1
NFYA
SP3
E2F1
SP1
MYC
ASCL1
POU5F1
Protein
Protein family
Complex
Chemical/Molecule
Phenotype/Stimulus
Nucleus
Membrane
Up-regulates
Down-regulates
Physical interaction
Unknown
Direct
Indirect
Binding
